# Long COVID: the elephant in the room

**DOI:** 10.1093/qjmed/hcab299

**Published:** 2021-11-26

**Authors:** B D Kelly, G Gulati

**Affiliations:** From the Department of Psychiatry, Trinity College Dublin, Trinity Centre for Health Sciences, Tallaght University Hospital, Tallaght, Dublin 24, D24 NR0A, Ireland; Schools of Law & Medicine, University of Limerick, Limerick, Ireland

The COVID-19 pandemic and long COVID present the greatest challenges to health and mental health services in a generation. How we respond to these tests will determine the future of clinical care and the quality of life of millions of people for many years to come.

As the pandemic continues, the neuro-psychiatric effects of COVID-19 and the psychological impact of public health restrictions are increasingly apparent both in the population at large and among specific groups. Populations of particular concern include people infected with COVID-19, healthcare and other frontline workers, and people whose lives and livelihoods are impacted by necessary public health restrictions.^1^^,^^2^ Many people fall into more than one of these risk groups; e.g. healthcare workers who contract COVID-19 and cannot work owing to long COVID.

People who are infected with COVID-19 present particular cause for concern in terms of mental health and restoring social function. The lingering psychological and psychiatric effects of acute infection are increasingly clear. One meta-analysis of COVID-19 survivors 14 or more days after recovery from the acute infection found alarmingly high rates of persistent psychological distress (36%), anxiety disorders (22%), depression (21%) and post-traumatic stress disorder (20%).^3^ More than one-third of survivors (35%) had sleeping disorders, which further diminish mental health, physical wellbeing and social function.

Another retrospective cohort study of 236 379 survivors of COVID-19 used electronic health records to examine neurological and psychiatric outcomes over 6 months.^4^ In this analysis, the estimated incidence of a neurological or psychiatric diagnosis over 6 months was 33·6% for all COVID-19 patients, rising to 46.4% for those who had been admitted to an intensive therapy unit (ITU). Anxiety disorders, in particular, were diagnosed in 17.4% of all COVID-19 patients and 19.1% of those admitted to ITU.

In this study, most neurological and psychiatric diagnoses were more common in patients who had COVID-19 compared to influenza (hazard ratio [HR] 1.4) or other respiratory tract infections (HR: 1.2). First episodes of mood, anxiety or psychotic disorders were substantially more common following COVID-19 than influenza (HR: 1.8), and the overall risk of psychosis was twice as high (HR: 2.0).

This study and the broader literature provide clear, consistent evidence that the psychological sequelae of COVID-19 infection are common, substantial and sustained. From a population health perspective, these prolonged effects will likely multiply the overall suffering of COVID-19 patients several-fold. Moreover, we are still not clear how long these effects will last or what their further consequences might be; e.g. delayed functional recovery, impaired quality of life, persistent psychological impairment, self-harm or suicide.

For many survivors of acute COVID-19 infection, these psychological and neuro-psychiatric features form a key element of long-COVID syndrome.^5^ According to the National Institute for Health and Care Excellence (NICE), Scottish Intercollegiate Guidelines Network (SIGN) and Royal College of General Practitioners (RCGP), ‘post-COVID-19 syndrome’ is present when signs and symptoms that develop during or after an infection consistent with COVID-19 persist for more than 12 weeks and are not explained by an alternative diagnosis.^6^ There are usually clusters of symptoms, often overlapping, which tend to fluctuate and change over time, and can impact on any body system.

The term ‘long COVID’ is used to describe signs and symptoms that continue or develop after acute COVID-19, and includes ongoing symptomatic COVID-19 (from 4 to 12 weeks) and post-COVID-19 syndrome (12 weeks or more), according to the Consensus Recommendation in the NICE guidance.

Given that the condition is now formally recognized, how common is long COVID? One study identified post-acute COVID-19 syndrome in 50.9% of COVID-19 patients between 10 and 14 weeks after disease onset.^7^ Fatigue was very common (34.8%), along with diminished quality of life in survivors with post-acute COVID-19 syndrome (66.9%). Clearly, this is a condition in need of sustained attention, careful evaluation, multi-disciplinary treatment and ongoing study.

Inevitably, there are many unanswered questions. First, we require a more detailed understanding of the evolution of the symptoms of long COVID in order to better characterize the condition, identify specific treatments and determine the precise roles of neuro-biological injury, psychological determinants and other factors in its genesis and maintenance.^8^^,^^9^ Second, we need to understand to what extent people with undiagnosed, asymptomatic COVID-19 infection can develop long COVID, and how might this be detected and distinguished from other diagnoses. Third, more data are needed on the possible role of vaccination or specific treatments for COVID-19 in relation to long COVID; do they decrease risk, ameliorate the condition, or have some other impact that is as yet unclear?

Most urgently, we need multi-disciplinary treatment programmes to identify and manage the long-term sequelae of acute COVID-19 for patients who are currently affected by long COVID. These programmes can build on what is already known about other post-viral syndromes and gather new longitudinal evidence about long COVID in particular.^10^

We know that long COVID is an urgent, growing issue, so the time to act is now.

*Conflict of interest*. None declared.
